# Decadal-scale onset and termination of Antarctic ice-mass loss during the last deglaciation

**DOI:** 10.1038/s41467-021-27053-6

**Published:** 2021-11-18

**Authors:** Michael E. Weber, Nicholas R. Golledge, Chris J. Fogwill, Chris S. M. Turney, Zoë A. Thomas

**Affiliations:** 1 Institute for Geosciences, Department of Geochemistry and Petrology, University of Bonn, Bonn, Germany; 2 Antarctic Research Centre, Victoria University of Wellington, Wellington, New Zealand; 3 School of Geography, Geology and the Environment, University of Keele, Staffordshire, UK; 4 Earth and Sustainability Science Research Centre, School of Biological Earth and Environmental Sciences, University of New South Wales, Sydney, NSW Australia; 5 ARC Centre of Excellence in Australian Biodiversity and Heritage, School of Biological, Earth and Environmental Sciences, University of New South Wales, Sydney, NSW Australia

**Keywords:** Palaeoclimate, Cryospheric science

## Abstract

Emerging ice-sheet modeling suggests once initiated, retreat of the Antarctic Ice Sheet (AIS) can continue for centuries. Unfortunately, the short observational record cannot resolve the tipping points, rate of change, and timescale of responses. Iceberg-rafted debris data from Iceberg Alley identify eight retreat phases after the Last Glacial Maximum that each destabilized the AIS within a decade, contributing to global sea-level rise for centuries to a millennium, which subsequently re-stabilized equally rapidly. This dynamic response of the AIS is supported by (i) a West Antarctic blue ice record of ice-elevation drawdown >600 m during three such retreat events related to globally recognized deglacial meltwater pulses, (ii) step-wise retreat up to 400 km across the Ross Sea shelf, (iii) independent ice sheet modeling, and (iv) tipping point analysis. Our findings are consistent with a growing body of evidence suggesting the recent acceleration of AIS mass loss may mark the beginning of a prolonged period of ice sheet retreat and substantial global sea level rise.

## Abstract (web summary)

It may have taken only a decade to repeatedly destabilize the Antarctic Ice Sheet after the last Ice Age as shown by a new data-model study. The ice sheet lost ice for centuries each time before it abruptly re-stabilized again.

## Introduction

Deciphering past sea-level changes during times of natural climate warming can be useful for understanding the consequences of current and future human-induced climate warming. Here, the role of the Antarctic Ice Sheet (AIS) as the largest remaining ice sheet on Earth is crucial, yet remains poorly understood^1^. The future evolution of the AIS represents the largest uncertainty in sea-level projections of this and upcoming centuries^2^. The West Antarctic Ice Sheet (WAIS) holds an equivalent of 4.3 m global mean sea-level rise (GMSLR), of which 3.4 m rests on a bed below present sea level. The marine-based Wilkes Land basin of the East Antarctic Ice Sheet (EAIS), which holds an equivalent of 3–4 m GMSLR, also has sectors exposed to marine ice-sheet instability that have the potential to discharge ice for centuries to millennia^3^.

Basal ice-shelf melting induced by a warming ocean has been identified as a key trigger of marine ice sheet instability, leading to enhanced ice flow across the grounding line^4^. Invoking additional processes such as hydrofracturing and ice-cliff failure, AIS sea-level contributions could be up to ~1 m by the year 2100, and up to ~16 m by the year 2500 in higher CO_2_ emission scenarios^5^. However, recent reassessment shows that these mechanisms are not necessarily needed to explain geological and historical mass loss. Models that do not include them have a more modest GSLR projection for the 21^st^ century with an AIS contribution of just 10–20 cm, under representative concentration pathway 8.5^6–8^.

Geological evidence can provide the pace, timing and source of previous contributions from the AIS and thus offer a context for these predicted future changes. For the mid to late Holocene, sea level can be considered more or less stable, with decimeter-scale fluctuations^9^. The natural warming following the last Ice Age—the deglaciation—lasted from ~19,000 to ~8000 years ago and was accompanied by a GMSLR up to 134 m^10^. Sea level did not rise uniformly but in distinct Meltwater Pulses (MWP) such as MWP-19K, MWP-1A—when sea level rose ~16 m over ~360 years^11^—and MWP-1B. Since the AIS most likely contributed to these periods of rapid sea level rise, and since future AIS collapse could be substantial, approaching deglacial rates, studying processes of ice decay during the last deglaciation in pristine data archives can be extremely valuable to better understand the present state and future course of the AIS.

During deglaciation the AIS might have lost ice mass in response to three global environmental drivers or a combination thereof: subsurface ocean warming, atmospheric warming due to teleconnections to the equatorial Pacific, or sea-level forcing through Northern Hemisphere ice sheet mass loss. A warming subsurface ocean (in ~500–1500 m water depth) has been accepted as an important causal mechanism to destabilize the AIS in both the present^6,12,13^ and the past^14,15^. In this model, freshwater induced variations in deep-water formation regulate the Atlantic meridional overturning circulation and related heat transport into the Southern Hemisphere. As a consequence of reduced vertical mixing in the circum-Antarctic Southern Ocean, upwelling Circumpolar Deep Water does not lose its heat to the atmosphere as effectively, thereby intensifying the subsurface heat transport and intrusion beneath Antarctic ice shelves^16^, where it contributes to melting the AIS.

The surface mass balance of the AIS is influenced by atmospheric teleconnections such as the Southern Annular Mode^17^ and the El Nino-Southern Oscillation^18^ today. Although these relations are not well understood for the deglaciation, changes in tropical Pacific sea-surface temperatures could have induced changes in the surface mass balance of Northern Hemisphere ice sheets and thus were likely important for the growth to and retreat from their Last Glacial Maximum (LGM) extent^19^. For the AIS, however, such teleconnections would not lead to substantial changes in surface mass balance for the end of the LGM because of the different ice-sheet configuration and the colder climate, leading to a slightly positive ice-mass balance during ENSO-driven temperature increase because the threshold to thawing would not be crossed^20^.

It is well established that bedrock subsidence beneath the AIS and/or sea-level rise at its edge play a critical role in driving grounding-line retreat or prohibit AIS growth^21,22^, and conversely that a sea-level fall or bedrock uplift re-stabilizes the grounding line, reduces ice loss or promotes grounding line advance^23^. New ice-sheet models that are coupled to a global sea-level model show that AIS dynamics are amplified by Northern Hemisphere sea-level forcing^24^, suggesting a higher AIS volume during the LGM relative to models without this interaction, and a dynamic response of the AIS during deglaciation that is well in line with periods of rapid sea-level rise (MWPs) and the geological data sets presented here.

Despite the mounting evidence for present-day and future AIS instability, and increasing concerns about GMSLR, until recently there has been no individual record combining the pace, timing, and magnitude of past AIS mass loss to provide essential constraints for projections of the future AIS evolution. However, multiple AIS mass loss events for the deglaciation have recently been dated precisely using dust-climate synchronizations between marine cores and ice core cores (ref. ^14^, Methods). These events, termed Antarctic Ice Sheet Discharge (AID) events, lasted for centuries to millennia and are documented in deep-ocean sediment in the Scotia Sea’s Iceberg Alley^14^.

**Fig. 1:**
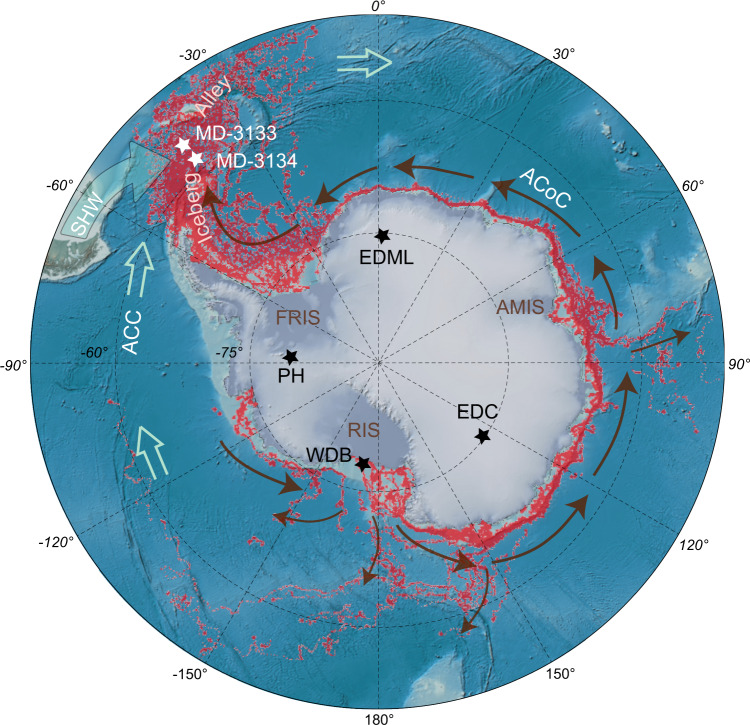
Location map and iceberg trajectories. Bathymetric map with DEM color shaded relief and Antarctic basemap (IBSCO/GEBCO08) (details at https://maps.ngdc.noaa.gov/viewers/bathymetry/). Sites MD07-3133 and MD07-3134 (white stars) are located in Scotia Sea’s Iceberg Alley. Red underlain pattern shows trajectories of large (≥5 km in length) icebergs calving off the Antarctic ice shelves between 1999 and 2009 (ref. ^25^). An animated version including iceberg trajectories from 1976 to 2019 was put together by PixleMoversAndMakers.com and can be viewed online (https://www.youtube.com/watch?v=iHySJkuoENk). Brown arrows indicate the general circum-Antarctic drift of icebergs that mainly follow the counterclockwise flow of the Antarctic Coastal Current (ACoC). Floating ice shelves are labeled in brown text (FRIS, Filchner-Ronne Ice Shelf; RIS, Ross Ice Shelf; AMIS, Amery Ice Shelf). Antarctic sites referred to in this study are shown by black stars (EDML, Epica Dronning Maud Land ice core; EDC, EPICA Dome C ice core; PH, Patriot Hills blue ice field; WDB, Whales Deep Basin, Ross Sea). Southern Hemisphere Westerlies (SHW) indicate Patagonia as main dust source and Iceberg Alley in its direct trajectory. The clockwise flow of the Antarctic Circumpolar Current (ACC) is indicated by open light blue arrows.

The majority of Antarctic icebergs route through Iceberg Alley after calving from the Antarctic margin^25^ and traveling counter-clockwise around Antarctica (Fig. 1). Melt rates remain low within the cold Antarctic Coastal Current until the warmer Antarctic Circumpolar Current is reached, after which icebergs ablate rapidly and release their iceberg-rafted debris (IBRD) in this iceberg cemetery. Although most of the coarser debris is released from sediment-laden basal ice close to the grounding line, fine-grained, englacial IBRD travels far and is the dominant size fraction (1–2 mm in diameter) found in Iceberg Alley sediment sites. Since icebergs account for approximately half of the total AIS mass loss^26^, the IBRD record from Iceberg Alley provides a sensitive, nearly continuous reconstruction of AIS dynamics by capturing an integrated signal of AIS mass loss^14,27^.

In this study, we primarily rely on published IBRD records from Iceberg Alley sites MD07-3133 and MD07-3134 and use the timing and magnitude of the deglacial AID events^14^ to decipher the pace of AIS destabilization and re-stabilization. However, instead of using the original age scale relying on the EPICA Dronning Maud Land (EDML) ice core, the newer AICC 2012 age scale^28,29^, which relies on interhemispheric methane correlation, was applied^24^. This age scale allows for direct comparison of climate events documented in ice cores from both hemisphere and has recently also been used to compare Northern Hemisphere ice-sheet events to our AID events^24^. Also, we carefully re-counted all IBRD transitions leading into, and out of, an AID event using the new chronology. For details concerning the age model, the stratigraphic resolution of the sampling, and the underlying uncertainties and IBRD counting procedures see ref. ^14^ and Methods.

We compare this offshore ice discharge record to published isotopic and chronological data from a blue ice field in the Patriot Hills, an area sensitive to ice-sheet thinning^30,31^, and to a novel, shallow-marine core transect across the Ross Sea shelf^32^ to infer the deglacial grounding line retreat history. We then use new model output from previous ice-sheet model experiments^6,15^ to examine the role and timing of calving in relation to total ice-mass loss. Finally, we analyze the IBRD records and ice-sheet model outputs for early signs of destabilization and re-stabilization using tipping point analyses.

## Results and discussion

### Decadal-scale onset and termination of deglacial ice-mass loss

**Fig. 2:**
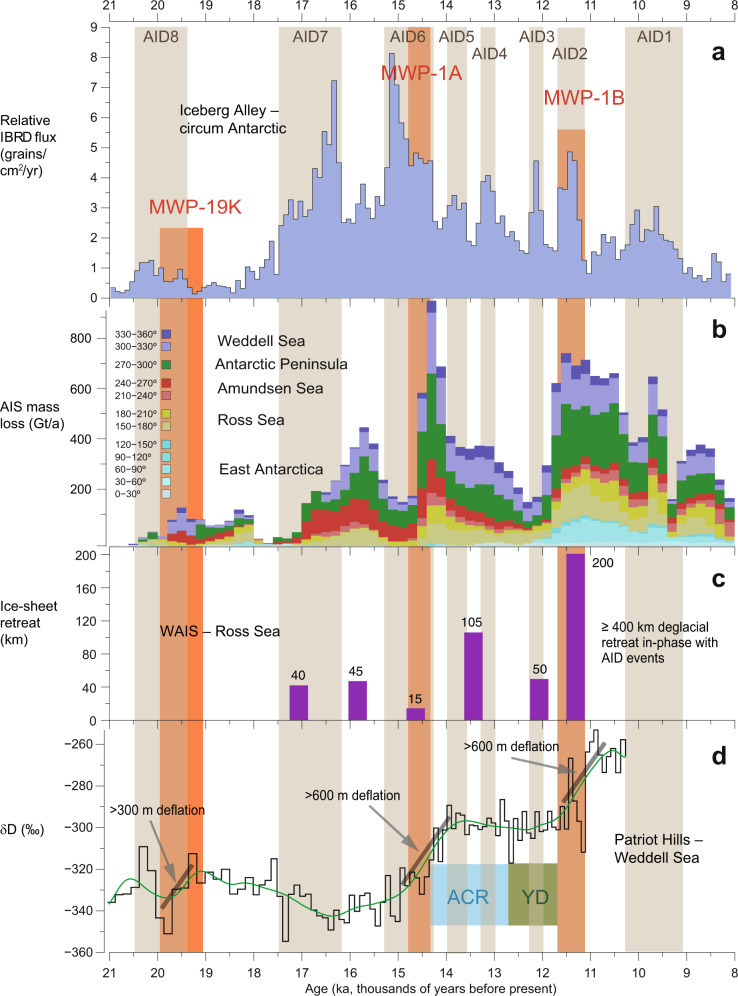
Data model comparison of deglacial ice-sheet and sea-level events. **a** Deglacial iceberg-rafted debris (IBRD) stack from Iceberg Alley^14^ with Antarctic Ice-Sheet Discharge events (AID) 1–8 on the AICC 2012 age scale^28,29^, averaged over 100-year increments, and the stratigraphic position of meltwater pulses (MWP)19K^19,20^, MWP-1A^11^, and MWP-1B^33^. **b** Antarctic-wide regional mass loss history from deglacial ice-sheet modeling experiments^15^, binned into 200-year intervals, and color-coded for different sectors of the AIS. Note that the values shown represent net mass loss from the ice sheet. **c** Deglacial retreat history of the WAIS, inferred from a core transect across the Ross Sea shelf^32^. **d** Deglacial elevation changes for the Weddell Sea sector of the AIS, inferred from δD isotope measurements on an ice core from the Patriot Hills^30^; black line denotes 2-point moving average; green line is smoothed. ACR Antarctic Cold Reversal. YD Younger Dryas. Note that simulated and empirical reconstructions show striking similarities (within the age uncertainties) with respect to AID events and global MWP, although all underlying individual age models were generated independently.

The sediment record from Iceberg Alley indicates multiple phases of enhanced iceberg routing during the last deglaciation that commenced and terminated abruptly, and lasted from centuries to a millennium^14^. Three out of eight AID events occurred at known times of global MWP events (Fig. 2): AID8 (~20–19 ka) as a precursor event around MWP-19K^19,20^; AID6 (~15–13.9 ka) constituting the peak of AIS deglaciation around MWP-1A^11^; and AID2 (~11.3 ka) at the time of MWP-1B^33^.

**Fig. 3:**
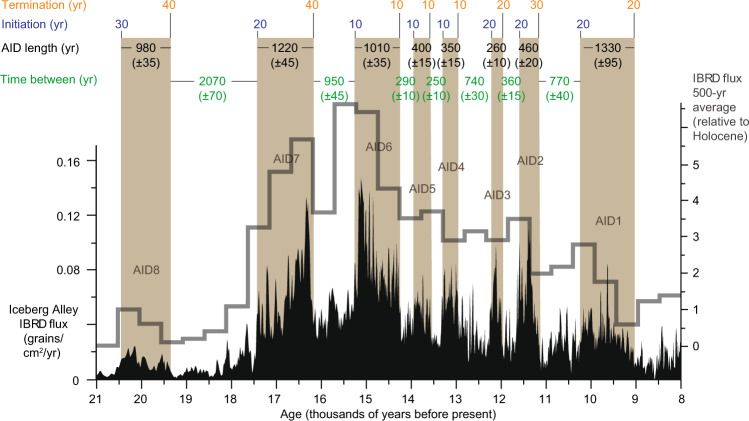
Pace and duration of AID events. Deglacial iceberg-rafted debris (IBRD) stack from Iceberg Alley with Antarctic Ice-Sheet Discharge events AID1–8, sampled in 10-yr increments at Sites MD07-3133 and MD07-3134 and stacked (black curve) as well as displayed relative to Holocene average in 500-yr increments (gray histogram) on the AICC 2012 age scale^28,29^. AID events 1–8 (brown bars) destabilized the AIS within 10–30 years (blue numbers), and continued to retreat for 250–1160 years (black numbers). Consecutive AIS re-stabilization took 10–40 years (orange numbers), and remained for 190–2090 years (green numbers). Numbers in parenthesis indicate 2-sigma error for the duration of AID events. Note that the true pace of AIS destabilization might be less than a decade based on higher-resolution Site MD07-3133 (see Methods).

The age model^14^ on the new AICC 2012 chronology^28,29^ (Supplementary Fig. 1) allows for tight constraints on the timing, pace, and duration of deglacial ice-sheet retreat. AID events from Iceberg Alley indicate periods of substantially enhanced ice-mass loss, which lasted 250–1160 years (Fig. 3) with uncertainties of 20–190 years for their duration^14^, respectively. The stable intervening periods lasted 190–2090 years with uncertainties of 20–140 years, respectively. Interestingly, the duration of the eight deglacial phases of ice-sheet destabilization inferred from AID events (~710 years on average; ~5700 years in total) is about the same as the duration of deglacial ice-sheet re-stabilization (~680 years on average; ~5400 years in total).

In light of the new chronology, the IBRD record was counted again for the start and end of all AID events to establish how quickly major changes in AIS mass loss happened. The results show that AID events initiated and terminated extremely rapidly (blue and orange numbers in Fig. 3, respectively), indicative of the threshold behavior typically found in ice sheets^34^. In most cases the flux of debris increased from low background levels to high values within a single sample and the decrease occurred equally rapidly. These rapid transitions from low to high and high to low IBRD contents form the basis of our manually assigned AID event classification. The counting interval was 1 cm, which translates into 8–17 years resolution for AID1–8, depending on the time interval and core. For core MD07-3133, the resolution was ~8 years/cm for most parts of the deglaciation, whereas it was mostly ~14 years/cm for core MD3134. Considering AID1–8 of the stacked IBRD record, which represents a larger-scale, regional response to ice-sheet changes, it took, on average, 17 years to initiate AIS mass loss, and 22 years to re-stabilize the AIS again (Fig. 3). Analyzing the two sites individually, which indicates local ice-sheet changes and avoids potential mismatches in the stacking of the records, reduces these average numbers to 13 and 12 years, respectively. Therefore, to decipher the true pace of change, we rely on Site MD07-3133 because of the higher sedimentation rate. Here, the 8-year sample resolution indicates that the majority of AID events took a decade or less to initiate and terminate. This implies that it could have taken substantially less than a decade to accelerate and slow down mass loss from the AIS during deglaciation and that the true pace of change could only be addressed with an even higher sedimentation rate site. We should add that relative uncertainties of only several decades for the multiple century-long AID events^14^ lend further credit to the robustness of the above numbers.

Interestingly, the decadal-scale onset and termination of AIS mass loss is consistent over all eight deglacial AID events, regardless of changing environmental, sea-level, and grounding line conditions. The recognition of such rapid AIS dynamics is an important novel finding that is relevant for the interpretation of recent abrupt changes in the AIS discovered by satellite observations and modeling studies.

We should note a significant lag time between iceberg release from the AIS and deposition in Iceberg Alley is unlikely. Also, there is no a priori reason that the time span between iceberg release and IBRD deposition should have varied substantially through the deglaciation because the major processes involved show little sensitivity to climate variability, i.e., the positions of the Antarctic Circumpolar Current (ACC), the Antarctic Coastal Current (ACoC), and the Antarctic Divergence as well as the Coriolis Force. Also, important mechanisms such as ocean thermal forcing or sea-ice variability do not affect iceberg transport significantly. Hence, these present-day processes likely operated in a more or less similar manner during deglaciation. For further information see ref. ^14^ and Methods.

### Ice core and marine core evidence from the West Antarctic Ice Sheet

Knowledge of the response of the AIS to past climate changes was previously based on only a few near-field, short sediment sequences, which limited our understanding of the dominant feedbacks between the AIS, Southern Hemisphere and Northern Hemisphere climate, and global sea level. Most shallow marine and terrestrial Antarctic sequences cannot be dated adequately, reveal only the final stage of ice retreat, or resolve only local responses to climate forcing, therefore leaving much room for speculation. Such shallow marine and terrestrial archives pointed to a rather stable AIS that mainly deglaciated late, in post-MWP-1A to late Holocene times with minor contributions to the deglacial GMSLR (e.g., refs. ^35,36^). However, our recognition of a highly dynamic AIS that deglaciated early and substantially starting with AID 7 at ~17 ka, is in line with an Antarctic-wide deglacial warming and retreat of sea-ice inferred from ice cores^37^ as well as enhanced upwelling in the Southern Ocean^38^ and a general southward displacement of the Southern Hemisphere Westerly winds^39^. In addition, we find clear evidence for early deglaciation in novel and pristine archives on the Antarctic shelf and continent.

First, the Patriot Hills blue ice field, for instance, forms a locally sourced compound glacier system (i.e., with negligible inflow) that is buttressed by the Institute Ice Stream via the Horseshoe Valley Trough. Ice-sheet modeling demonstrates that this area is highly sensitive to dynamic ice-sheet changes across the broader Weddell Sea Embayment and provides an independent measure of AIS surface elevation change^30,31^. Isotopic data and chronostratigraphic constraints of the Patriot Hills outcrop (Methods) suggest ice elevation drawdown of 300–400 m for AID8 (MWP-19K; Fig. 2d), 600–800 m for AID6 (MWP-1A), and again 600–800 m for AID2 (MWP-1B). AID7, a substantial ice-mass loss event 17–15.5 ka during early deglaciation, is not documented in the Patriot Hills record and was hence unlikely sourced from the Weddell Sea Embayment; neither is there evidence for most of the younger AID events (5–3).

Second, the first well-constrained chronology for deglacial grounding line retreat for the eastern Ross Sea shelf was developed from sediment wedges in Whales Deep Basin. Carbonate shell dating on a core transect revealed a step-wise retreat of the WAIS largely in-phase with AID7 to AID2 (Fig. 2c), exhibiting only minor retreat at AID6 (MWP-1A) and the largest individual retreat (~200 km) around AID2 (MWP-1B), with a total deglacial ice-sheet retreat in the Ross Sea of ~400 km^32^. The Ross Sea records suggest ice-margin changes across the Antarctic during AID events, most noticeably for AID6 and AID2 and associated MWP-1A and MWP-1B, respectively.

Third, ice-sheet models for the deglaciation^15^ simulate AIS ice mass change in response to changes in air and ocean temperatures, precipitation, and global mean sea-level. Although no specific iceberg calving mechanism was included, the temporal phasing of AIS mass loss arising from the combination of applied environmental forcings and independent age determination bears extremely close correspondence to the phasing, shape, and relative magnitudes of IBRD fluxes recorded in Iceberg Alley^14^ (Fig. 2a, b). For example, in both the IBRD and the model data, AID7 shows a gradual increase over a millennium, followed by a more abrupt decrease. AID6 exhibits a very abrupt initiation with a more gradual reduction to AID4.

The fact that AID events occur during times of the three global deglacial MWPs (Fig. 2a) that are partially also depicted in the ice-sheet model (Fig. 2b), the eastern Ross Sea retreat history (Fig. 2c), and the Patriot Hills elevation drawdown (Fig. 2d) clearly argues for a step-wise AIS contribution to global GMSLR. Considering that the overall age uncertainties in deglacial chronologies are up to a millennium (Supplementary Fig. 1), these comparisons suggest near-synchronous ice-margin changes across many sectors of the AIS, at least during the main meltwater pulses, although the magnitude of change may have varied substantially from region to region.

### Ice-mass loss at the grounding line—a modeling perspective

**Fig. 4:**
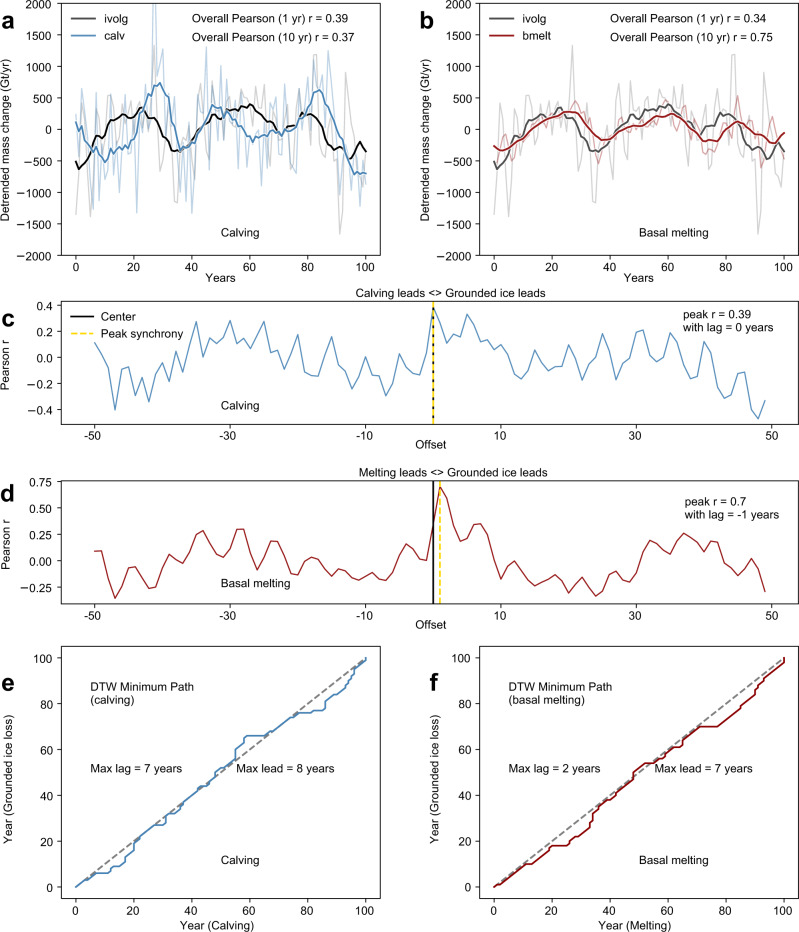
Modeled relation between iceberg calving, sub-shelf melt, and change in grounded ice mass. **a** Correlation between linearly detrended annually resolved and domain-integrated changes in modeled rate of calving loss and the rate of change of the volume of grounded ice (faint lines), and the same analysis for a 10-year filtered timeseries of the same data (bold lines). **b** The same analysis, but showing correlations between ice shelf basal melt rate and grounded ice volume. Faint lines show annual data, bold lines show 10-year filtered data. Note that colored lines in **a** and **b** give rate of change (in Gt/yr) and refer to total volume of grounded ice (ivolg), calving flux (calv), and flux from sub-ice shelf melting (bmelt). **c** Correlation coefficients for calving and **d** melting for a range of lead and lag magnitudes, up to 50 year. **e** Dynamic time warp (DTW) analysis showing time-varying offset between strongest point-wise correlations of calving or **f** melting with ice sheet volume change.

Changes in IBRD flux recorded in Iceberg Alley should be indicative of changes in calving rate and hence closely relate to the timing of mass-loss events of the grounded ice sheet^14^. To test this, we make use of AIS simulations that were systematically optimized to produce rates of calving, basal melt, surface mass balance, and total mass loss that reproduce observations through the 40-year satellite observational period^6^. This robustly constrained model now allows us to confidently explore the temporal relationship between iceberg calving and the rate of loss of grounded ice, because it reliably captures each of the key mechanisms controlling ice sheet mass balance. Furthermore, we can do this at annual resolution. We first calculate correlation coefficients between modeled rates of calving and rates of grounded ice volume change, and between basal melting and grounded ice volume change. This is performed on linearly detrended annual outputs, and on a 10-year filtered version of these (Fig. 4a, b). Calving appears to be (slightly) more strongly correlated with grounded ice volume change at 1 year than 10 year, whereas basal melting has a much stronger correlation at 10 year. To investigate whether leads or lags exist between grounded ice mass loss and rates of calving or basal ice shelf melting we next calculate global correlations (that is, a single measure of the correlation between the entire span of each set of two timeseries being compared, rather than for a particular subset of the data) for integer lead/lag values from zero up to half the timeseries length (50 year). For calving the best fit is zero lag (Fig. 4c). For basal melting, there is a much stronger relationship if mass loss actually precedes melt by 1 year (Fig. 4d), most likely because increased mass loss leads to thicker or more extensive ice in the floating domain, producing higher total basal melt. Finally, we test whether the apparent synchrony based on global correlations could be obscuring time-varying offsets. Using a dynamic time warp (DTW) analysis to calculate the shortest distance between points in the correlated datasets (Fig. 4e, f) shows that the best fits between calving and ice-sheet loss, and melting and ice sheet loss, are all close to the 1:1 line, and where they deviate the maximum offsets are all less than a decade.

From these analyses we are able to conclude with some confidence that changes in the rate of grounded ice loss are tightly linked at annual to multi-annual frequencies to both calving and basal melting. In the context of the approximately decadal resolution of our IBRD record, therefore, we find that iceberg calving events on decadal time scales effectively reflect synchronous discharge of grounded ice from the AIS.

### AIS mass loss estimation for MWP-1A based on IBRD flux rates

Antarctica is losing ice mass through calving of icebergs as well as surface and basal melting. The estimated total iceberg calving flux from Antarctica is ~1300–2000 Gt/yr^40^, with giant icebergs—those larger than 18 km in length—representing at least half of the total AIS mass loss (~1100 Gt/yr)^41^. More recent estimates report calving rates of ~1300 Gt/yr, with equal mass loss from melting^26^, or 1100 Gt/yr with slightly higher relative melt rates^42^. In a steady-state system, this annual ice mass loss will be compensated by snowfall on land or basal freezing, a condition that can be assumed for the AIS during the mid to late Holocene and during the LGM; both rather stable ice-sheet periods when the IBRD flux rates remained relatively low. However, during deglaciation, the IBRD flux increased distinctly—up to nine times above the low average during MWP^14^ (see right scale in Fig. 3 showing that even the 500-year IBRD flux averages are at least six times higher during MWP-1A than during the Holocene).

Since an annual ice mass loss of an additional 1000 Gt/yr would lead to ~2.6 mm of GMSL/yr, the IBRD flux record implies that, over the course of MWP-1A for instance, which lasted a little less than 400 years, ~9 m of GMSL could have originated from the AIS at average annual calving rates of 1300 Gt/yr (ranging between 7 and 11 m, depending on the chosen annual AIS calving rates of 1000 or 1500 Gt/yr^42^, respectively). These are maximum numbers since the actual contribution to GMSLR will be due only to the fraction of ice that is above flotation thickness and therefore sea-level relevant. Based on ref. ^15^, ~32% of ice mass delivered to the ocean corresponds to ice above the flotation thickness. Hence, the global mean sea-level contribution of the AIS to MWP-1A could be at least 2.5–3.5 m. Due to uncertainties these are rough estimates only; however, they indicate that a notable fraction of MWP-1A’s 16 m GMSLR could have originated from Antarctica.

### Ice-sheet behavior indicates critical tipping point

The abruptness of the initiation and termination of the AID events suggests that there may be thresholds that, once passed, could trigger a destabilization or re-stabilization of the ice sheet. If these tipping points are irreversible in the sense that there is hysteresis—that is a direct reversal of the forcing does not allow a return to the previous state—certain ‘early warning indicators’ may identify some characteristic fluctuation patterns present in a timeseries preceding an abrupt change. These patterns form due to the increasingly slow return to equilibrium from perturbations as a critical threshold is approached, termed ‘critical slowing down’^43,44^. We investigated whether these AID events might be characterized by a critical threshold, whereby early warning signals of the impending abrupt change might be detected (Methods).

**Fig. 5:**
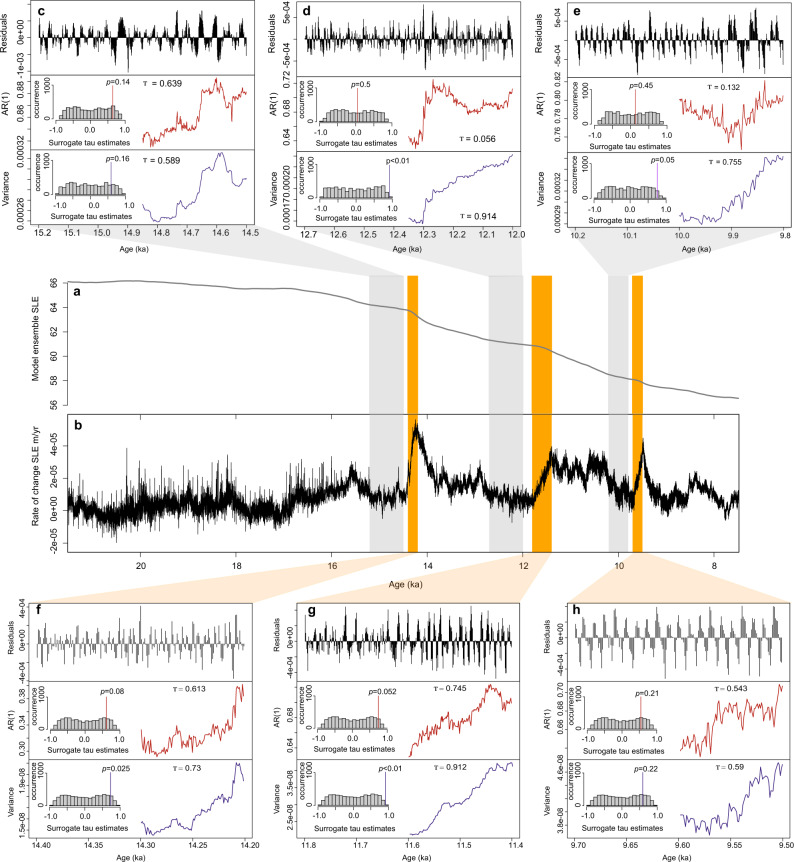
Tipping point analysis for deglacial model simulations. **a** Model ensemble of sea-level equivalent (SLE) mass loss (m/yr) and **b** SLE rate of change (m/yr). Orange and gray columns depict data selected for analysis of destabilization and re-stabilization, respectively. Tipping point analysis for the data prior to destabilization (**c**–**e**) and data prior to re-stabilization (**f**–**h**) of the AID-like event, from top: data residuals, autocorrelation (red) and variance (purple), over a 50% sliding window. Histogram showing Kendall tau correlation coefficients for surrogate time series, and corresponding *p* values for autocorrelation (red lines) and variance (purple lines).

We first analyzed the Scotia Sea IBRD stack, looking at data prior to an AID event (to analyze early warning signals for destabilization) and during an AID event (for re-stabilization). Although some AID events displayed increases in autocorrelation and variance prior to the abrupt change, most did not (Supplementary Figs. 2–3), and similar results were found for the unstacked records. There are a number of possible explanations for this. Proxy data may not always faithfully record early warning signals due to the lower resolution of the data, high noise level, short data length, and by virtue of being a proxy rather than direct measurement of ice sheet stability^45^, which can mask the patterns of critical slowing down. Alternatively, the signals may not have been recorded due to their absence, either because there is no critical transition, or if the system ‘tipped’ before reaching the critical threshold. To try to resolve this we undertook the same analysis on the model simulations for sea-level equivalent (SLE) mass loss (Fig. 5a). Previous studies have successfully detected early warning indicators of critical transitions in model simulations of Antarctic mass loss, using the same techniques as described here^46,47^. Three phases of substantial ice mass loss corresponding, within the dating uncertainties, to AID6 (MWP-1A), AID2 (MWP-1B), and AID1 were identified in our simulations. We found an increase in autocorrelation and variance, leading indicators of critical slowing down^48^, prior to both the destabilization and re-stabilization phases of the simulated AID events (Fig. 5).

Our analysis indicates that a conceptual model for Antarctic ice sheet hysteresis^49^ is a useful framework, with positive and negative feedbacks modulating the pulses of ice mass loss for each AID event. Recent modeling studies suggest that relatively abrupt local re-stabilization of the AIS after an ice-mass loss event can be caused by rapid bedrock uplift, providing a negative feedback for further grounding line retreat^50,51^, although it is currently unclear if this process is applicable to the timescales of AID re-stabilization reported here^52^.

**Fig. 6:**
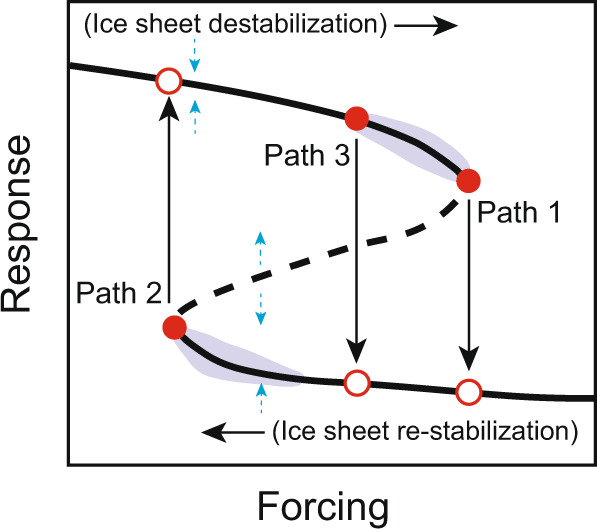
Schematic stability landscape for ice-sheet destabilization and re-stabilization pathways. Stability diagram showing non-linear responses to forcing. Black solid lines indicate stable equilibria; black dashed line indicates an unstable equilibrium; small blue arrows denote the direction in which the system moves when it is not on the curve (and therefore not in equilibrium). Pathway 1 (‘destabilization’) and pathway 2 (‘re-stabilization’) both show a non-linear change associated with a critical transition (i.e., from red filled circle to red hollow circle). Pathway 3 (‘destabilization’), shows ‘early escape’ with a tipping point occur prior to reaching the critical threshold. Purple shaded area indicates areas where early warning signals may be detected.

Our IBRD data suggest that this uplift feedback would need to have a re-stabilization effect within a decade. Positive feedbacks for destabilization include the ice-elevation feedback and marine ice sheet instability^2,5,42,45^, and likely contribute to the rapid onset of the AID events. In many simulations of Antarctic mass loss, the forcing is applied very slowly to allow equilibrium to be maintained in order to detect hysteresis branches^49,53^. In practice, however, it is likely that for destabilization, the ice sheet ‘tips’ well before reaching the actual critical threshold (Fig. 6, path 3), due to the speed of the forcing and the external ‘noise’ or perturbations, known as ‘early escape’^54,55^. In this case, early warning indicators are likely to fail. However, in studies using observational data which are higher time resolved, early warning signals of ice sheet destabilization have been detected^56^, indicating that the lack of better time resolved data from marine sediment cores may potentially explain the failure to detect early warning signals. These issues have important implications for detecting future rapid destabilization of ice sheets using statistical indicators, notably, that high-resolution data and appropriate model forcing is likely necessary.

Local destabilization could cause a complete disintegration of the marine ice in West Antarctica^57^. Under this model scenario, the region disequilibrates after 60 years of currently observed melt rates. Our data from Iceberg Alley suggests that such disequilibration could happen faster, within a decade or two. If a destabilization in the Amundsen Sea sector is initiated, Antarctica is projected to irrevocably contribute at least 3 m to GMSLR on centennial- to millennial-scales^57^, consistent with our data that documents centennial to millennial-scale iceberg discharge following each destabilization. Other modeling studies lend support to this pattern of events by showing that long-term GMSL commitments can be triggered by much shorter-lived (decadal to centennial-scale) ice-shelf thinning or collapse^5,58–60^. According to our combined reconstruction-modeling study of past extreme events, the negative mass balance of the AIS in recent decades^61^ and especially the rapid retreat of parts of the WAIS^62^ might very well constitute a novel AID event, marking the onset of self-sustaining and irreversible ice-sheet retreat for centuries.

## Methods

### Cores and samples from Iceberg Alley

Sample-based investigations concentrated on deep-sea cores retrieved in the Scotia Sea during Marion Dufresne II cruise 160 in March 2007. Sites MD07-3133 (57°26’S, 43°27’W; 3101 m water depth; 32.8 m long) and MD07-3134 (59°25’S, 41°28’W; 3663 m water depth; 58.2 m long) originate from the northern end of Dove Basin and Pirie Bank, respectively.

### Chronology of Iceberg Alley sites

The original chronology and AID records of sites MD07-3133 and MD07-3134 have been published before^14,63^. The age model of the Scotia Sea sites is based on low-resolution ground-truth data sets associated with Marine Isotopic Stage (MIS) boundaries and a high-resolution age model relying on distinct dust-climate couplings between Southern Ocean sediment and the Antarctic EPICA Dronning Maud Land (EDML) ice core^14^. The Scotia Sea sites lie downwind from the major dust source (Patagonia) but in closer proximity than Antarctic ice cores (Fig. 1). Age models built upon dust proxy data sets such as magnetic susceptibility as well as Ca and Fe records measured through X-ray fluorescence, present a major step forward in developing Southern Ocean chronologies for the Pacific, the Atlantic, and the Scotia Sea (ref. ^63^ and references therein) providing clear evidence for coherent and synchronous changes in dust deposition across the Southern Ocean and the AIS^14^.

The age model of Site MD07-3134 consisted of 48 control points based on dust proxy tie points. For the last deglaciation, five confident tie points relative to EDML exist for the period ~20 to ~8 ka, which covers 10 m and 7 m core length for Sites MD07-3133 and MD07-3134, respectively. The five tie points correspond to consistent breaks in slopes and characteristic and reproducible peaks in the dust proxy records of MS and Ca relative to ice-core dust proxy non-sea salt Ca^2+^ (nssCa)^14^. All in all, the published tie points were sufficient to achieve synchronicity between the marine and ice-core record during deglaciation, especially during the time ~19–13 ka, when the main decline in atmospheric dust occurred.

To allow for hemispheric and interhemispheric comparison of ice-sheet events, the newer AICC 2012 age scale^28,29^ was developed based on methane correlation. Accordingly, all ages of the IBRD record have been transferred from EDML1 to AICC 2012 (ref. ^24^). This new age scale makes ages progressively older further back in time. The difference is minimal for the mid to late Holocene relative to EDML1. At the time of MWP-1B the difference is on the order of 150 years, whereas it is 350 years during MWP-1A and ~500 years at the LGM, before which the differences become smaller again (Supplementary Fig. 1). The variable age offset is the main reason to re-count the transitions in and out of AID events. The implication of this age shift, however, is that AID events 8, 6, and 2 remain within the timing of MWP-19ka, 1A and 1B, respectively. Therefore, the conclusions we draw hold regardless of the age model applied.

### Age scale uncertainties

Since atmospheric dust transport can be considered to be geologically instantaneous, no major leads or lags should be present between ice core and ocean core. In addition, the Iceberg Alley cores are deep-ocean sites with continuous, undisturbed deposition of mud, indicating that the deposits are in stratigraphic order. Even if sedimentation rates may vary between tie points, it should only be gradual given the rather homogenous lithology, and it does not affect the average sample resolution between tie points. Moreover, the fact that most physical and geochemical properties can be correlated between Sites MD07-3133 and MD07-3134 on a cm-scale argues for only minor disturbance by sediment bioturbation and attests that sediment focusing (i.e., introduction of material from lateral sources) happened syn-sedimentary with minor effect on the preservation of signals.

The original age uncertainty estimates of the Scotia Sea sites^63^ built upon a bootstrap algorithm that simulates possibly wrong TPs and therefore provides conservative error estimates for each AID (see Extended Data Fig. 2 of ref. ^14^). Resulting dating errors (2-sigma) of the tie point correlation were used to calculate uncertainties for the start, end, and duration of AIDs. All uncertainties are incorporated into the absolute dating uncertainty (2-sigma error) of the AIDs. The bootstrap algorithm allows for the derivation of relative dating uncertainties for the AIDs. Since interpolation errors of nearby layers are highly correlated, these uncertainties are naturally smaller than the absolute dating uncertainties. Hence, significantly more precise estimates were made on the duration of and the distance between AID events. These durations are at least several centuries with relative uncertainties of only a few decades (Fig. 1) that can be considered robust (see discussion in ref. ^14^). However, the absolute age uncertainties of EDML1 age model range from 400 to 1000 years between 8 and 21 ka.

For the AICC 2012 age scale, we considered full uncertainty estimates by propagating the square roots of the uncertainties from the tie point correlation and the uncertainties of the AICC 2012 age scale and marked those for the upper and lower bounds of each AID event (Supplementary Fig. 1; see also ref. ^24^). The results reiterate that AID events 8, 6, and 2 occurred, within uncertainties, during the timing of MWP-19ka, 1A and 1B, respectively, and that no neighboring AID event would overlap enough to indicate otherwise. Any leads or lags between MWPs and AID events can, however, not be established due to the uncertainties.

### IBRD size and counting

The sediment matrix of Sites MD07-31333 and MD07-3134 ranges from fine-grained mud (typical for the LGM) to diatom-bearing mud to diatom oozes (typical for the Holocene). Individual small IBRD (usually 0.5–2 mm in diameter) are loosely embedded in this continuously deposited, fine-grained matrix. IBRD counting was conducted every cm on x-radiographs taken from 1-cm thick and 10-cm wide slices that were cut out from the center of each core segment and exposed to an X-ray system. 0–2 IBRD grains within a 1-cm interval would be considered low background values. However, slightly higher counts (≥5 grains, for instance) can already translate into an AID event if neighboring counts are elevated, too. Even high counts typical for peak iceberg discharge during an AID event rarely exceed 10 grains per 1-cm interval, translating into 1 debris grain only that is loosely embedded in a muddy sediment matrix of 1 cm^3^. This illustrates that as little as one IBRD grain can represent an individual iceberg that could have traveled very far before melting in the substantially warmer waters of the ACC (see below). Such loose IBRD packing is markedly different from Northern Hemisphere Heinrich Events, where IBRD is usually packed in discrete layers that consist almost entirely of lithic grains 0.1–3 mm in size (ref. ^64^).

The classification into AID events has been made by eye. In a first step, we defined events using the transitions from low to high IBRD content for both sites independently. In a second step, the distinction has been repeated for IBRD flux for both sites independently to find the best compromise between content and flux records. In a third step, the classification has been applied to the stacked record to find the best compromise for a regional record consisting of both sites rather than local records^14^.

### The Patriot Hills horizontal ice core and age model

A new ‘horizontal’ ice core (1600 m) has been extracted from exposed ancient ice from within Horseshoe Valley (Ellsworth Mountains; 80°18′S, 81°21′W). The site is located 50 km inland from the modern grounding line of the Filchner-Ronne Ice Shelf in the Weddell Sea Embayment (WSE), making the area sensitive to dynamic ice-sheet changes across the broader region^30,31^.

The chronological framework for the Patriot Hills Blue Ice Area (BIA) has been developed using a combination of volcanic (tephra) horizons and trace gas samples. The record presented here includes new data that builds upon that presented in ref. ^30^, and utilizes Bayesian modeling to integrate the chronological control points through the record. The trace gas measurements provide a range of possible age solutions, which together with the absolute constraint provided by the tephra horizons, allows the development of a robust chronological framework that can be tied directly to the isotopic profile through high-resolution ground-penetrating radar survey^65^. This can be tested against the ages of independent geochemically typed tephras identified in the BIA profile^30^. Geochemical analysis confirms the interpretation of WCM-93-25 (ref. ^66^) (18.2 ± 5.8 ka) as PH279, and further analyses suggests the Trachyte PH282b correlates to TD822a (17.61 ± 0.73 ka)^67^ with a similarity coefficient of >0.953.

We undertook Bayesian age modeling using the software package OxCal v.4.2.4 using a Poisson process deposition model (P_sequence)^68,69^ to integrate the above accepted gas measurements with the tephra ages. Using Bayes theorem, the algorithms employed sample possible solutions with a probability that is the product of the prior and likelihood probabilities. Crucially, Bayesian modeling enables the relative stratigraphic information from the BIA transect to be incorporated along with the ‘calibrated likelihoods’ or ‘calibrated probability distributions’.

‘Calibration curves’ were developed for the three trace gas species using the same datasets used above^70^ with 20-year resolution. Each gas species was given a Delta_R term to allow for uncertainty between the measurements at Patriot Hills and those reported in the calibration curves^70^. The Delta_R term was calculated using the ‘f’ and ‘t’ terms. The ‘f’ variable sets the default error on the measurements as a factor of the standard deviation in reported calibration curves; the ‘t’ value sets how tight the tails are on the Delta_R. Our age model used a ‘f’ value of 0.3 and a ‘t’ value of 2 degrees of freedom, allowing the value to be further from the mean than a Normal distribution. Taking into account the deposition model, the ages provided by the tephras and the common age solutions offered by the trace gas measurements, the posterior probability densities quantify the most probable age distributions. To account for the gas-ice age difference (Δage), we used a Delta_R term for the different sections of the BIA, guided by recently reported values from the WAIS ice core^71^. For the glacial age ice, we used the prior U (−500, −200) and the Holocene, U (−300, −150). For the glacial age ice, we found a mean Δage of 350 ± 87 year; for the Holocene we determined a Δage of 225 ± 43 year.

δD and δ^18^O isotopic measurements were performed between 1 and 3 m resolution at James Cook University (JCU) using Diffusion Sampling - Cavity Ring-down Spectrometry (DS-CRDS) (International Atomic Energy WICO Lab ID. 16139) (ref. ^31^). This system continuously converts liquid water into water vapor for real-time stable isotope analysis by laser spectroscopy (Picarro L2120-i, Sunnyvale, CA, USA). To ensure reproducibility, a subset of samples was rerun at UNSW ICELAB for δD and δ^18^O using a Los Gatos Research Liquid Water Isotope Analyser 24d (International Atomic Energy WICO Lab ID. 16117). Reported overall analytical precision on long term ice core standards is <0.32‰ for δD and <0.13 for δ^18^O values. All isotopic values are expressed relative to the Vienna Standard Mean Ocean Water 2 (VSMOW2). Given the isolated nature of Horseshoe Valley both during contemporary times and deglaciation, and the buttressing effect of the AIS on ice flow from the valley, we interpret the isotopic trend captured in the Patriot Hills BIA as the result of ice-sheet elevation changes due to mass loss across the broader WSE. Thus, increasing δD and δ^18^O water isotope values across the ACR and the apparent local warming can only reflect regional ice-sheet draw down.

### Modern versus deglacial AIS mass loss and IBRD deposition

Today, icebergs take several months to two years to travel counterclockwise around Antarctica^40^ within the Antarctic Coastal Current (ACoC) (Fig. 1). Although icebergs lose some mass in transit, for instance, through wave erosion, friction, or collisions with sea ice, the rapid transport in cold waters can be considered geologically instantaneous. Melt rates remain low until the warmer Antarctic Circumpolar Current (ACC) is reached, after which the icebergs ablate rapidly^72^ in Iceberg Alley close to our core sites. Some smaller icebergs may stray directly north due to topographic steering of ocean currents (smaller brown arrows in Fig. 1), but even the majority of the smaller icebergs stay entrained in the ACoC. Also, there is a regional bias, i.e., more proximal sources (Weddell Sea and East Antarctic Ice Sheet) supply more icebergs than more distal sources in Wilkes Land or the Ross Sea. However, Iceberg Alley is the main gateway for Antarctic icebergs to exit high southern latitudes, supporting the notion that only here IBRD provides a direct measure of Antarctic ice-mass loss through iceberg calving, whereas similar interpretations for other Antarctic and open Southern Ocean regions, e.g. ref. ^73^, may be difficult to reconcile because other debris transport processes such as sea-ice and ocean currents^74^ may be more important or even dominate the ice-rafted signal.

There are several factors supporting the notion that iceberg routing during the deglaciation was not much different from today. First, the Antarctic Divergence is the major driver of the ocean-atmospheric circulation in the Southern Ocean. It forces winds north of it to blow east and to feed clockwise into the ACC, which is geographically constrained to 50–55°S (ref. ^75^). This is specifically true for the Drake Passage, which is a major bottle neck for the ACC east of our core sites. On the other hand, winds blow west and feed counterclockwise into the ACoC around Antarctica south of the Antarctic Divergence. These currents are large planetary geostrophic currents that mainly operate independent of climate. The ACC and ACoC will thus likely exist under different climates of the past^39,76^, and although individual fronts connected with them likely shifted north or south in response to changing sea-ice coverage and climate, these shifts can be considered minor directly east of the Drake Passage.

Second, the Coriolis force is important for the routing of icebergs after calving from the AIS. This force is also independent from the state of climate because it depends on the rotation of earth. It causes all Antarctic icebergs to be deflected to the left, move counterclockwise and maintain a course very close to the edge of the Antarctic continent. In addition, the Coriolis force exerts a greater force on larger icebergs^77^, which contribute to the majority to AIS mass loss, and they thus largely remain entrained in the ACoC (i.e., sea ice does not have a strong effect on altering the course of large icebergs); although smaller icebergs and crawlers may have escaped the ACoC during times of thicker sea-ice coverage farther away from the continent.

Third, several studies have reconstructed extensive coastal polynyas immediately in front of the AIS during colder periods when sea-ice coverage was dense and reached farther away from the continent^20,78^. This further implies a vigorous ACoC also during glacial and deglacial times.

Fourth, ocean thermal forcing has been proposed as primary mechanism to explain distinct phases of AIS mass loss during deglaciation^14,15,30,79^. However, the requisite temperature increase would be subsurface warming of Circumpolar Deepwater in ~500–1500 m water depths, i.e., lies deeper than the typical draughts of drifting icebergs. Therefore, ocean thermal forcing could induce enhanced ice melt at the grounding line at times of increased resumption of the Atlantic Meridional Overturning Circulation but this would not affect drifting icebergs.

### Ice-sheet modeling

For this study, we re-analyzed outputs from previous ice-sheet simulations^6,15^. Our deglacial ice-sheet model^15^ employed the Parallel Ice Sheet Model (PISM) at 15-km resolution for transient ice-sheet/ice-shelf simulations from the LGM to present. The age models in these simulations are completely independent from the IBRD age models. Full methodological details are provided elsewhere, but in summary, PISM calculates evolution of the three-dimensional thermal and flow characteristics of the ice-sheet using a combination of the shallow-ice/shallow-shelf approximations of the Stokes equations^80^, and a novel enthalpy scheme^81^. Fast-flowing grounded ice evolves in areas where modeled basal porewater pressure increases and thereby reduces basal substrate shear strength, following the plastic till failure approach^82^.

Ice-mass loss is assumed to take place through calving of icebergs and sub-ice shelf melting. Although no transiently evolving calving margin is accommodated in our simulations, we nonetheless impose a fixed calving front at the continental shelf break by removing ice seaward of the −2000 m bathymetric contour. This is justified on the grounds that contiguous floating ice of a thickness less than the depth of the continental shelf (approximately 1000 m) is unlikely to survive in the open Southern Ocean. Ice-mass lost through this mechanism changes through time as a consequence of changes in ice thickness reaching the calving front, but the margin itself does not migrate. Given that the change in the length of the calving front during deglaciation is small relative to its total length, we anticipate that this limitation of our model should not substantially affect the results presented here. Sub-shelf mass flux in our model is prescribed according to a timeseries scalar of zonal mean subsurface (approximately −400 m) ocean temperature evolution from transient deglacial ocean-atmosphere simulations using the LOVECLIM intermediate complexity model^16^. We apply this timeseries forcing to all floating ice in the domain using sub-ice shelf mass flux rates that are scaled to allow both LGM and present-day grounded ice extents to be reproduced^15^. Optimization experiments revealed that different scalings of effective ‘melt rate’ were required for each of the four ocean thermal forcings used, but all resulted in rates in the range 0–20 m/yr. During episodes of rapid ocean warming such as MWP-1A, thermal forcing of 0.5–1 °C translates to an equivalent change in sub-ice shelf mass flux of -10 m/yr. Bedrock deformation due to ice thickness changes are accounted for using the native elastic lithosphere, relaxing asthenosphere model of PISM (e.g. ref. ^83^). This model allows the long-term viscous effects of ice loading to be calculated and bedrock elevation adjustments to take place through the model simulations in a way that in previous experiments allowed present-day uplift rates measured from in situ Antarctic global positioning system receivers to be well captured^84^. Due to limitations of the 1-dimensional earth model, however, we are only able to implement single values for parameters that affect earth deformation across the entire domain. We use a lithospheric density of 3300 kg/m^3^, a flexural rigidity of 5 × 10^24^ Nm, and a mantle viscosity of 1 × 10^21^ Pa s. Our model does not capture changes arising from self-gravitational or earth rotational effects.

Environmental boundary conditions such as global mean sea level, air temperature, and subsurface ocean temperature are set using proxy-based and model-derived time series perturbations to present-day gridded datasets. Our ensemble of experiments combines three different sea-level reconstructions with four different ocean temperature curves, yielding twelve discrete ice sheet simulations. Parameter optimization and iterative adjustment of the scaling of the applied ocean forcing allow the thickness evolution of the ice-sheet from 25–0 ka to reproduce the spatially variable pattern of elevation changes indicated by twelve Antarctic ice core records. Marine geological data are used to constrain the horizontal extent of the simulated LGM ice sheet. The age scales used for each of these forcings, as well as their respective temporal uncertainties, differ, however. In presenting the ensemble mean pattern of Antarctic mass loss, therefore, we are unfortunately not able to reproduce the precise absolute timing of ice mass change revealed by the IBRD record. For this reason, we focus primarily on noting the correspondence between the pattern and rate of changes in each dataset, rather than the chronological synchroneity.

Ice-sheet modeling experiments used to investigate the temporal relationship between calving and grounded mass loss rates (Fig. 4) make use of simulations in which individual mass loss components (surface mass balance, basal mass balance, and calving flux) are constrained by observational data^6^. The verified mass loss partitioning in those experiments thus allows reliable inferences to be made regarding the link between calving (leading to IBRD deposition) and mass loss from the ice sheet.

### Tipping point analysis

Generic rules have been developed that can identify certain ‘early warning indicators’ to identify the characteristic pattern of fluctuations present in data preceding a non-linear change^43,48,85^. These patterns, manifested as an increase in autocorrelation and variance over a sliding window, form due to the increasingly slow response of the system to perturbations as a critical threshold is approached, termed ‘critical slowing down’. Procedures involving measuring the indicators of critical slowing down are described in detail in refs. ^47,48,86^. The data is pre-processed by removing long-term trends using a loess filter and interpolated (if necessary) to create an equidistant timeseries. Autocorrelation and variance (using the R functions acf() and var(), respectively) are then measured over a sliding window of size 50% of the data length. To provide a quantitative measure of the strength of the trends, the nonparametric Kendall’s tau rank correlation coefficient is applied^48,87^ to each individual time series. Significance tests provide a quantitative assessment of whether indicator trends are robust, by varying certain parameters such as the loess smoothing span and size of the sliding window. A null model is created by randomization of the dataset over several thousand permutations, which guarantees the same amplitude distribution as the original time series but destroys the linear correlation and structures. The statistical significance of the original analysis is obtained by comparing against the probability distribution of the null model. Specific sections of data from the Scotia Sea IBRD stack were selected to investigate potential thresholds for destabilization and re-stabilization. Sections of data for analysis were chosen to include only data prior to the re-stabilization/destabilization event. To investigate whether the stacking procedure had an effect on the results, we also applied the same analysis on data from the individual sites MD07-3133 and MD07-3134. Selected data from the deglacial ice-sheet model simulations were also analyzed; these data have a higher data resolution, lower noise level, and constitute a more direct proxy to the stability of the ice sheet.

## Supplementary information

Supplementary Information

## Data Availability

The datasets generated for this publication are available in the PANGAEA database (10.1594/PANGAEA.937485)^88^.
