# Supplementary material for: Decadal-scale onset and termination of Antarctic ice-mass loss during the last deglaciation
Source: Nat Commun. 2021 Nov 18;12:6683. doi: 10.1038/s41467-021-27053-6 (PMC8602255; doi:10.1038/s41467-021-27053-6)
Supplement: Supplementary file 1 — Supplementary Information [file 41467_2021_27053_MOESM1_ESM.pdf]

Supplementary Information for

Decadal-scale onset and termination of Antarctic ice-mass loss during the last deglaciation

Michael E. Weber (1)*, Nicholas R. Golledge (2), Chris J. Fogwill (3),
Chris S.M. Turney (4,5), and Zoë A. Thomas (4,5).

(1) Institute for Geosciences, Department of Geochemistry and Petrology, University of Bonn, Germany.
(2) Antarctic Research Centre, Victoria University of Wellington, New Zealand.
(3) School of Geography, Geology and the Environment, University of Keele, Staffordshire, UK.
(4) Earth and Sustainability Science Research Centre, School of Biological Earth and Environmental Sciences, University of New South Wales, Australia.
(5) ARC Centre of Excellence in Australian Biodiversity and Heritage, School of Biological, Earth and Environmental Sciences, University of New South Wales, Australia.

Contents

Supplementary Figures 1–3
Supplementary References 1–4

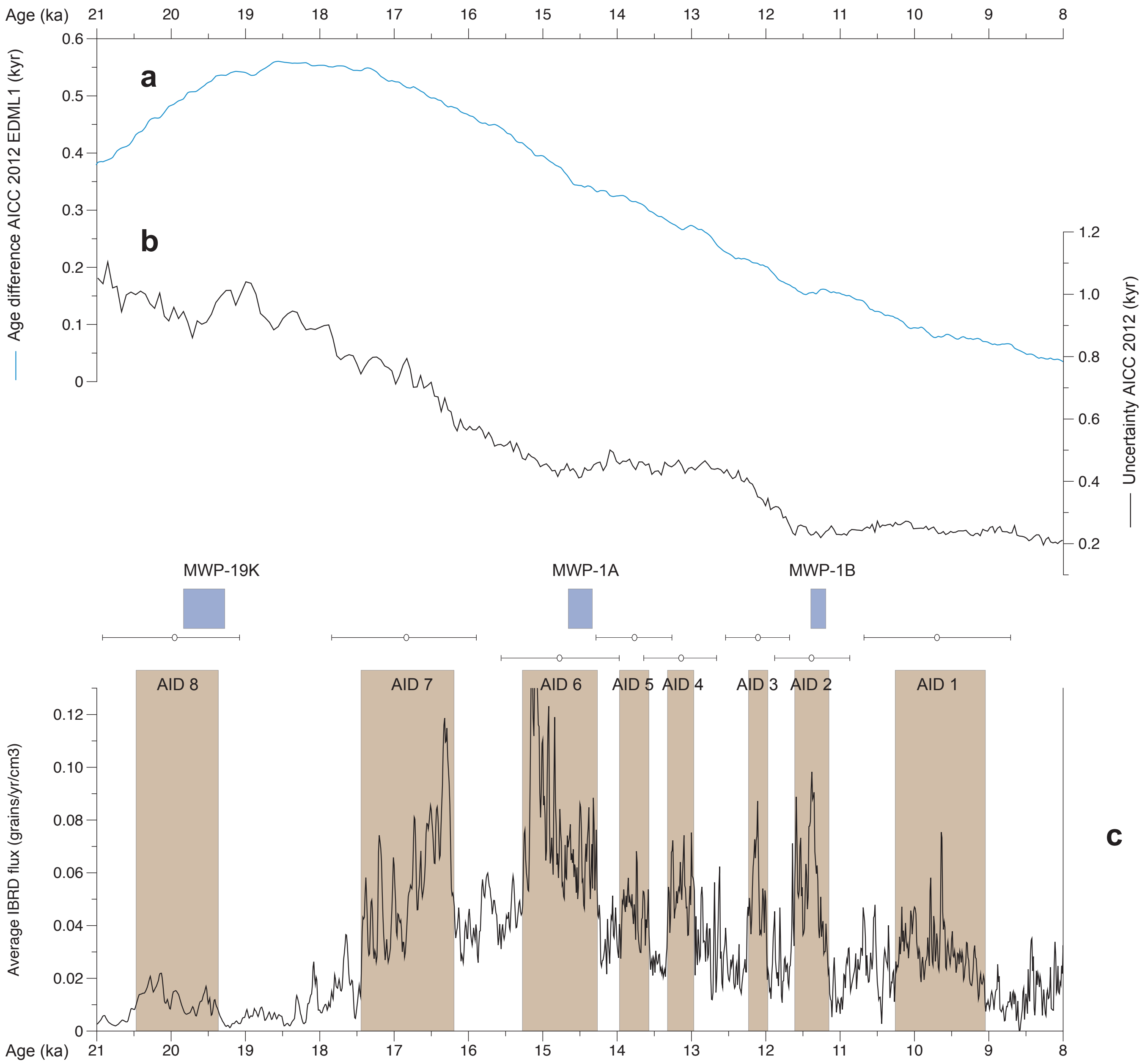

Supplementary Fig. 1. Uncertainties of AID events on the AICC2012 age model. a Age difference between the EDML1 (ref [1]) and AICC 2012 (ref [2,3]) age scales. **b** Uncertainties of the AICC 2012 age scale. **c** AID events 1–8 (brown bars) on the AICC 2012 age scale with age uncertainties for the upper and lower bounds on top. Note that deglacial meltwater pulses (MWP, blue rectangles) 19K, 1A and 1B occurred, within uncertainties, during AID8, 6 and 2, respectively. Note that 8–18 ka is from[4] and 18–21 ka is added in this study to accommodate AID8.

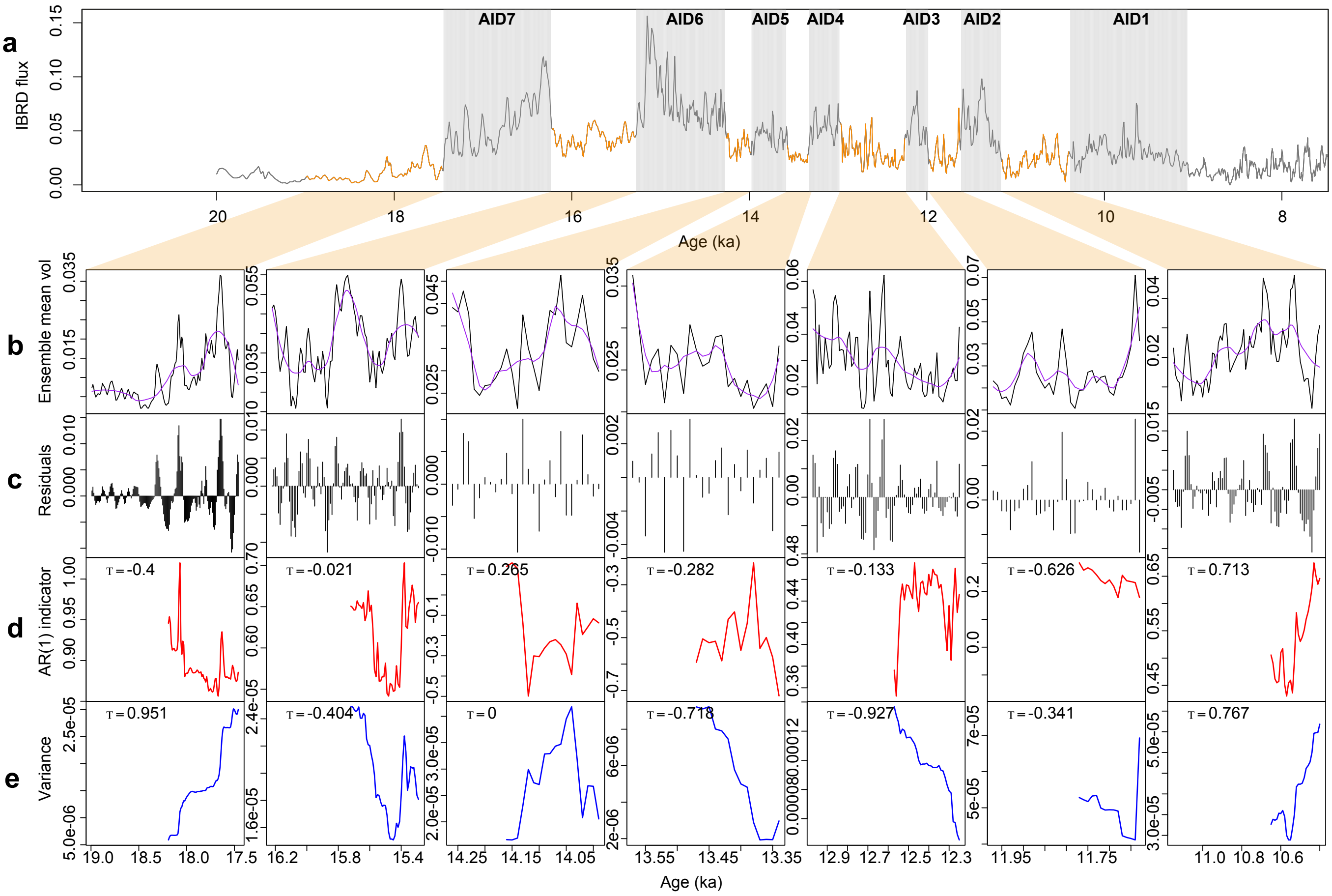

Supplementary Fig. 2. Tipping point analysis from the Iceberg Alley IBRD stack for destabilization pathway. a Scotia Sea IBRD stack AID events (gray columns) on AICC 2012 chronology (ref [2,3]), orange lines show data selected prior to each destabilization (AID) event. **b-e** Tipping point analysis for the data prior to destabilization, from top panel: loess detrending line (purple) from ensemble mean ice volume (black); data residuals (black); autocorrelation (red); and variance (blue), over a 50% sliding window, with Kendall Tau rank correlation coefficient labelled.

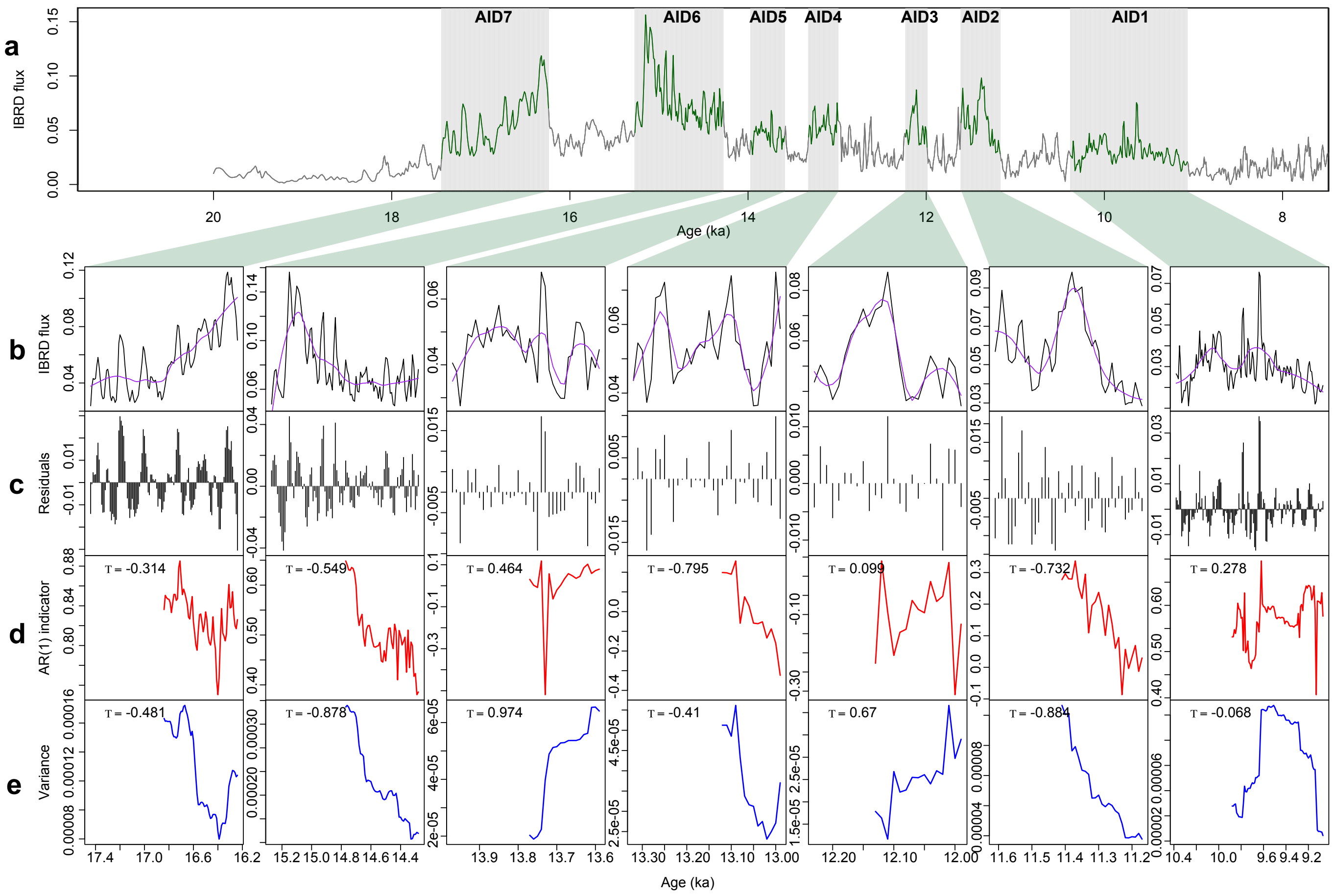

Supplementary Fig. 3. Tipping point analysis from the Iceberg Alley IBRD stack for re-stabilization pathway. a Scotia Sea IBRD stack with AID events (grey columns) on AICC 2012 chronology (ref [2,3]), green lines show data selected during each AID event, prior to re-stabilization. **b-e** Tipping point analysis for the data prior to re-stabilization, from top panel: loess detrending line (purple) from ensemble mean ice volume (black); data residuals (black); autocorrelation (red); and variance (blue), over a 50% sliding window, with Kendall Tau rank correlation coefficient labelled.

Supplementary References

1. Ruth U*, et al*. "EDML1": a chronology for the EPICA deep ice core from Dronning Maud Land, Antarctica, over the last 150 000 years. *Climate of the Past* **3**, 475-484 (2007).

2. Bazin L*, et al*. An optimized multi-proxy, multi-site Antarctic ice and gas orbital chronology (AICC2012): 120–800 ka. *Clim Past* **9**, 1715-1731 (2013).

3. Veres D*, et al*. The Antarctic ice core chronology (AICC2012): an optimized multi-parameter and multi-site dating approach for the last 120 thousand years. *Clim Past Discuss* **8**, 6011-6049 (2012).

4. Gomez N, Weber ME, Clark PU, Mitrovica JX, Han HK. Antarctic ice dynamics amplified by Northern Hemisphere sea-level forcing. *Nature* **587**, 600-604 (2020).
